# Northern Bobwhite roost site selection influences survival during the non-breeding season

**DOI:** 10.1007/s00442-026-05952-z

**Published:** 2026-08-07

**Authors:** Justin N. Hill, Emily B. McCallen, James A. Martin

**Affiliations:** 1 Warnell School of Forestry and Natural Resources, University of Georgia, Athens, GA 30602 USA; 2 Indiana Department of Natural Resources, Division of Fish and Wildlife, Bloomington, IN 47401 USA

**Keywords:** Northern bobwhite, Ideal free distribution, Resource selection, Roost, Survival

## Abstract

A key assumption of the ideal free distribution theory is that animals select resources based on environmental cues that maximize fitness, thereby facilitating natural selection within a population. Habitat management and conservation practices are commonly based on results of resource selection models with the assumption that selection and fitness are correlated. This is concerning given that this critical assumption has been demonstrably violated in certain systems, where animals select resources that ultimately reduce vital rates. We studied how nocturnal habitat selection of 6 common landcover types influenced survival rates of Northern Bobwhite (*Colinus virginianus*) on public lands in southern Indiana, USA during the non-breeding season. We found that selection for woody cover had a positive effect on survival rates. Our results suggest that ideal free distribution is facilitated in this system by minimizing predation risk through the use of woody roosting cover. To develop more comprehensive knowledge on habitat selection for species, studies should examine the effects that selection has on one or more vital rates.

## Introduction

Understanding the distribution of individual animals and the fitness consequences of resource use are key concepts in ecology. Furthermore, identifying attributes within the environment that contribute positively to population-level fitness is paramount to conserving species of interest. Many studies in this ilk rely heavily on the assumption of Fretwell and Lucas’s (1969) ideal free distribution (IFD) where animals distribute themselves among habitat patches that maximize fitness. Under this theory, animals use environmental cues (biotic and/or abiotic) to inform their decision on what patch to select. It is assumed under IFD that individuals have complete knowledge of their surroundings and can move freely from one patch to another (Fretwell and Lucas 1969). Broadening upon this concept, individuals would be presented with tradeoffs regarding predation risk and density driven competition for resources, such as food or mates (Brown and Kolter 2004; DeCesare et al. 2014). Under the assumptions of IFD, selection should be correlated with fitness (Manly et al. 2002), thereby facilitating natural selection over time. However, if sought after resources that confer fitness are limited, individuals that don’t have access to these resources will suffer negative fitness consequences.

Ecological trap theory has shown contradictory evidence regarding IFD’s core principle that selection coincides with fitness (Dwernychuk and Boag 1972; Gates and Gysel 1978; Delibes et al. 2001; Donovan and Thompson 2001). Ecological traps occur when individuals select resources based on environmental cues that make them attractive, yet the use of this resource has negative impacts on fitness. Oftentimes, management and conservation practices are based on results of resource selection models, even though we know that the assumptions of IFD may not always hold true. Without a more in-depth understanding of how resource selection influences vital rates and population viability, situations may arise where conservation actions preserve ecological traps by promoting resources that are pernicious to wildlife.

Despite a corpus of literature on animal resource selection, studies relating resource selection to fitness remains rare (Spencer 2002; Bloom et al. 2013; DeCesare et al. 2014; Long et al. 2016; Sinnott et al. 2021; Munar-Delgado et al. 2024). The Northern Bobwhite (*Colinus virginianus*; hereafter, bobwhite), an upland gamebird endemic to North America, is perhaps one of the most-studied species in the world, yet studies of this nature are mostly absent. Bobwhite habitat selection has been well examined across multiple behavioral states and life-history stages, including seasonal third-order selection (McGrath et al. 2017), nesting (Taylor et al. 1999), foraging (Hill et al. 2023), and brood rearing (Palmer et al. 2012), all of which may influence survival, as predation is the primary mortality risk throughout the bird’s lifecycle (Burger et al. 1994; Lunsford et al. 2019). Despite the vast body of research conducted on bobwhite, knowledge on habitat requirements during nocturnal times remains limited. This is especially concerning since bobwhite spend ~ 40–50% of their time roosting during the fall and winter. Concerns regarding population declines have led to numerous habitat selection studies (Yoho and Dimmick 1972; Martin et al. 2009; Janke and Gates 2013; Rosche et al. 2019; Lappin et al. 2023), where the collection of demographic rates (e.g., survival, nesting success, fecundity) would be ascertainable in many cases due to direct observations permitted by tracking equipment (King et al. 2006; Rader et al. 2007; Fuller and Fuller 2012; Klaassen et al. 2014). However, only one study, to our knowledge, has examined the effects of habitat selection on bobwhite survival (Sinnott et al. 2021). Within the limited body of literature on nocturnal ecology, it appears evident that selection varies with age, season, and scale (Wiseman and Lewis 1981; Perkins et al. 2014; Kubečka et al. 2021; Hill et al. 2023), and that predation risk likely influences it (Hiller and Guthery 2005; Perkins et al. 2014; Hill et al. 2023).

**Fig. 1:**
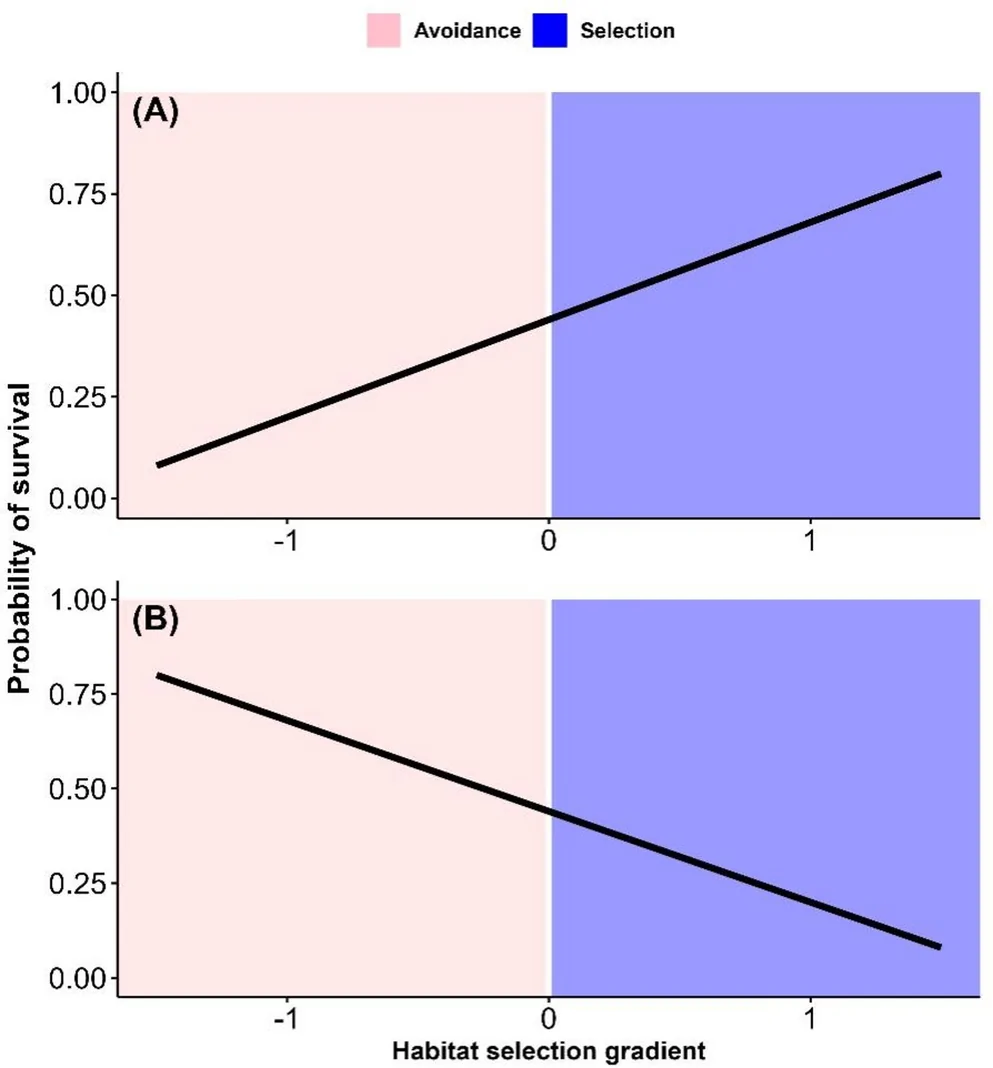
Hypothetical depiction of ideal free distribution (**A**), where vital rates have a positive interaction with resource selection rates, and an ecological trap (**B**), where vital rates exhibit a negative interaction along the resource selection gradient

We radio-tagged and tracked wild bobwhite on a public land site in southern Indiana, USA for two years during the non-breeding season to collect habitat use and survival data. We had two objectives: (1) determine the influence of multiple landcover types on nocturnal habitat selection during the non-breeding season and (2) determine if, and to what degree, nocturnal habitat selection of specific landcover types influenced survival during this season. The first hypothesis (not competitive with hypotheses two and three) we tested was that predation risk would influence habitat selection. We used 6 landcover types, as proxies of predation risk, to estimate effects of roost site selection. We predicted that bobwhite would avoid landcover types associated with greater predator densities or limited ground level vegetation that may aid in predator concealment during nocturnal times. We predicted that roosting birds would select cover types that were associated with lower predator densities and areas with vegetation structure conducive for vigilance and predator evasion. Germane to landcover types, we predicted that roosting birds would avoid woodlands, wetlands, and row-crop fields, and select grasslands, disturbed areas (burned grasslands/fallow fields), and areas close in proximity to woody cover (shrubs and tree saplings). The second (ideal free distribution) and third (ecological trap) hypotheses we tested by examining how individual nocturnal habitat selection influenced survival. Under the ideal free distribution hypothesis, habitat selection should coincide with fitness benefits. We predicted that selection of beneficial cover types would have positive effects on survival (i.e., behavior is adaptive, Fig. 1A). In contrast, under the ecological trap hypothesis, we predicted that nocturnal habitat selection would negatively affect survival (i.e., behavior is maladaptive, Fig. 1B).

## Materials and methods

### Study site

**Fig. 2:**
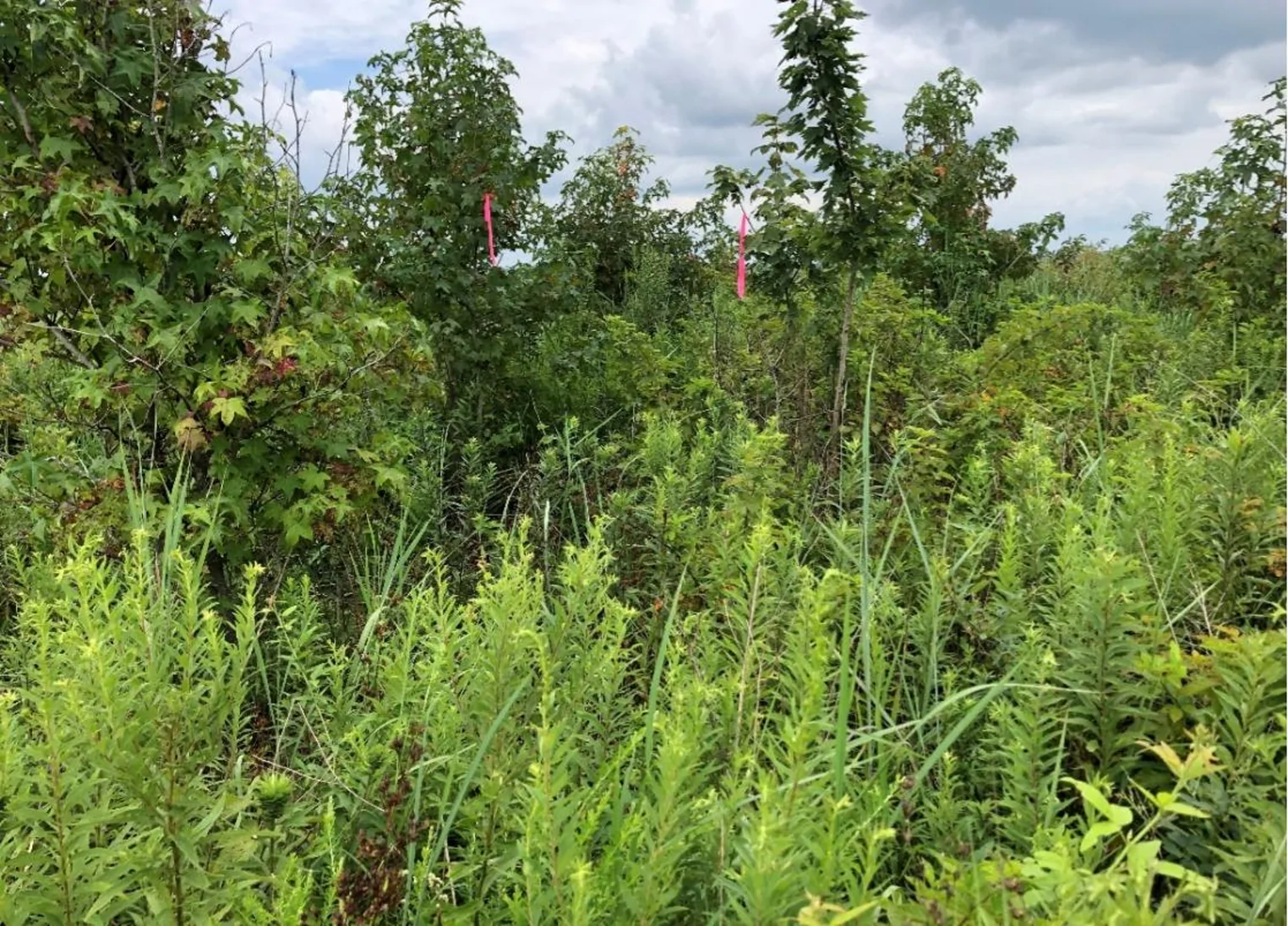
Example of a typical woody cover (early successional woody vegetation) patch located in a grassland at Goose Pond Fish & Wildlife Area, Greene County, Indiana, USA. Primary woody vegetation species pictured are sweetgum (*Liquidambar styraciflua*) and *Rubus* (spp.)

Our study site was Goose Pond Fish and Wildlife Area (GPFWA); located in Greene County, Indiana, USA. The property was owned and managed by the Indiana Department of Natural Resources. The landscape that encompassed GPFWA (3,681 ha) was primarily composed of agricultural fields and pasture lands that historically provided habitat for northern bobwhite. GPFWA was predominantly composed of constructed wetlands at the central area of the property, with ~ 1200 ha of native warm-season grasslands (grasslands) and rotational row-crop fields situated on the peripheries of the site. Wetland water levels, and associated proportions of inundated land, varied seasonally dependent upon management objectives and precipitation. Grasslands varied in size (4–90 ha) and were maintained via prescribed fire, mowing, and disking. Most prescribed fire occurred during the dormant season, and fire return intervals ranged from one to four years. Grasslands burned in the dormant season were often homogenous with regards to plant structure and diversity, as most areas were dominated by dense stands of Indiangrass (*Sorghastrum nutans*), big bluestem (*Andropogon gerardii*), switchgrass (*Panicum vergatum*), and contained low densities of forbs and woody cover. Common species associated with woody cover patches were *Rubus* (spp.), Japanese honeysuckle (*Lonicera japonica*), or pioneering tree species such as winged-sumac (*Rhus copallinum*), sweetgum (*Liquidambar styraciflua*), red maple (*Acer rubrum*), black cherry (*Prunus serotina*), or ash (*Fraxinus* spp.). These species were typically 1–3 m in height. Forb diversity was greater in disked areas of grasslands, however, most disked sections accounted for small proportions of the grasslands. Row-crop fields (1–20 ha) production rotation was as follows: one-year corn, one-year soybean, two-years fallow. During the fallow period, row-crop fields were primarily composed of forbs and pioneer grasses. For grasslands and row-crop fields, woody cover was most commonly present in narrow (~ 1–10 m wide) linear strips in drainages, field borders/fence rows, and filter strips, but did persist in small patches (~ 5–25 m^2^) scattered throughout peripheries of grasslands which had not been burned for > 1 year (Fig. 2). Several areas of the property were designated as upland habitat improvement areas (1–3 ha), which were primarily early successional plant communities that were maintained with disking/herbicide and small food plots to create habitat for bobwhite. Each May, 8–10 Mourning Dove (*Zenaida macroura*) fields (2–4 ha) were planted in sunflower to provide hunting opportunities during fall. Dove fields typically had a high forb component on the outermost areas, providing potential habitat for bobwhite. Other portions of the property were composed of woodlands and strip pit mine reclamation areas. Woodlands varied with regards to structure, but were dominated by oak (*Quercus* spp.), hickory (*Carya* spp.), and ash (*Fraxinus* spp.) species. Most woodlands provided small amounts of bobwhite habitat, oftentimes only occurring on the outermost portions. Strip pit mine reclamation sites were mainly closed canopy eastern white pine (*Pinus strobus*) forests, which contain little to no understory and ground level vegetation except on the peripheries.

Bobwhite management was a secondary objective to waterfowl and other wetland associated avian species. Quail hunting was permitted bi-weekly at our study site but was regulated through a draw and quota system to limit hunting pressure. Prior to the hunting season (01-Nov–10-Jan), population estimates occurred for 5 sections of the property via covey counts. Quail hunting would close prior to the ending of the season for a respective unit if harvest quotas reached 30% of the estimated population.

### Data collection

We collected nocturnal habitat use and survival data for two fall/winter seasons during 2020-21 and 2021-22. From 01-Sept-2020 through 15-Oct-2020 and 1-Sept-2021 through 17-Oct-2021, bobwhites were captured via baited walk-in funnel traps (Stoddard 1931). We captured additional bobwhite at roost sites using spotlights and landing/mist nets from 16-Oct-2020 through 31-Oct-2020 and 13-Oct-2021 until 31-Oct-2021. Bobwhites weighing > 129 g were fitted with a 6 g Very High Frequency (VHF) necklace-style radio transmitter equipped with 24 h mortality switch (American Wildlife Enterprises, Monticello, FL, USA; Perdix Wildlife Solutions Ltd., Warwickshire, England). Sample sizes were determined via a larger harvest study, where an a priori simulation was conducted to determine the number of tagged bobwhites required to detect differences in seasonal survival across multiple experimental units. Overall trapping and tagging efforts were dictated by these experimental design constraints rather than being conducted opportunistically.

From October through February 2020-21 and 2021-22, we recorded nocturnal locations of radio-tagged birds one to three times/week from a distance of ~ 25–30 m via homing techniques (White and Garrott 1990). Locations were recorded between 45 min post-sunset and one hour pre-sunrise to ensure birds were at roost sites. Estimated locations of tracked bobwhite were recorded via GPS with high resolution (1–3 m) base maps.

We included six spatial datasets in our model which we believed would be influential to bobwhite nocturnal habitat selection during the fall/winter. These data were comprised of distance to wetland, distance to grassland, distance to row-crop fields, proportion of disturbed area, distance to woody cover, and distance to woodland. For the grassland dataset, we included the interior distance from edge which is reported as a negative distance (e.g., − 27 m is 27 m inside of the grassland). The disturbed landcover was composed of all areas burned the previous spring/summer, fallow the previous or current year, upland habitat improvement areas, and dove fields. We included strip pit mine reclamations into our woodlands landcover classification, as most suitable bobwhite habitat would only occur on the outermost regions of each cover type.

Our coarsest dataset resolution was 20 m^2^, so we resampled all spatial layers to this resolution using the Resample tool in ArcGIS Pro 2.5 (Esri, Redlands, CA, USA). To account for our maximum estimated telemetry error of 20 m, we used the Focal Statistics tool in ArcGIS Pro 2.5 (Esri, Redlands, CA, USA) with a 3 × 3 moving window on all spatial layers. To examine correlations amongst all spatial layers, we conducted a Pearson correlation coefficient test for each unique combination of datasets. All coefficient values fell between the range of − 0.7 to 0.7, indicating moderate to low correlation.

Three diurnal locations/week were also recorded for tagged birds to monitor survival from 1-Oct through 31-Mar 2020-21 and 2021-22. If the mortality switch was active for a radio-tagged bird, observers would locate and estimate mortality cause based on evidence at the kill site. If individual birds were separate from their respective group and remained in the same approximate location for two tracking occasions, despite no active mortality switch, observers would acquire a visual of the bird to ascertain if it was living or dead. All harvested birds were reported via the hunter during the day of the successful hunt.

### Habitat selection analysis

**Fig. 3:**
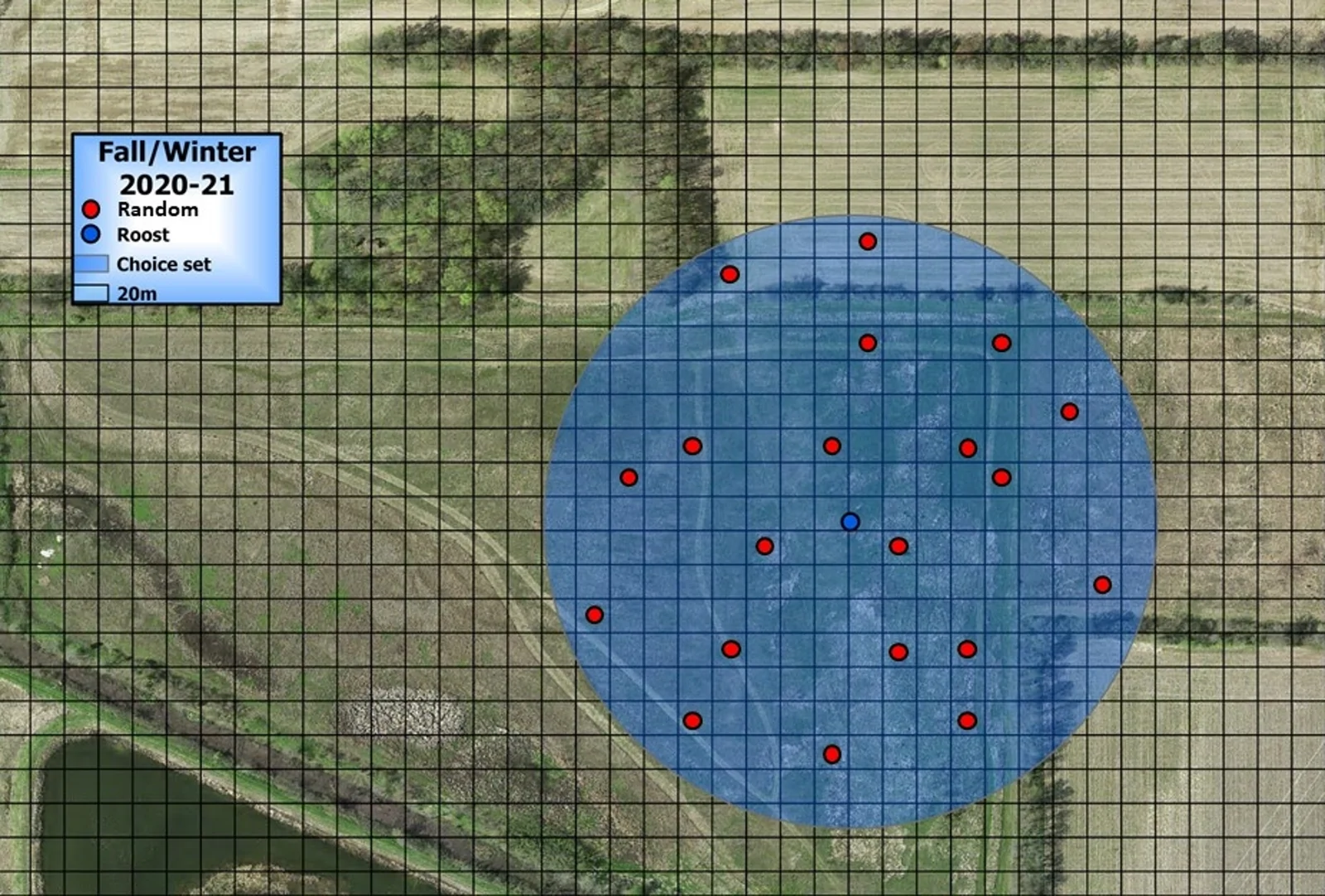
Example of a discrete choice-set used to sample use and available landcover variables for a unique roost site. Choice-sets were a circle centered on the used (roost site) location, with a radius of 180 m (~ mean distance between subsequent roost sites). A total of 20 randomly distributed locations within the choice set were sampled, as well as the used site. Resolution of all landcover variables sampled were 20 m^2^

We utilized a discrete choice sampling framework to determine use and availability of cover types at each roost site. This approach involved sampling the cover type attributes for the used and available sites within a unique choice-set (Cooper and Millspaugh 1999). Each choice-set was centered on the used roost site, while all available sites were within a 180 m radius of the roost. This distance was the mean distance between all subsequent roost sites for tracked bobwhite during the duration of the study. All spatial covariates were sampled using the extract function with the R “raster” package (Hijmans and Etten 2012) at the used site and 20 random sites within each choice-set (Fig. 3). We confirmed that 20 randomly sampled sites were an appropriate representation of available resources per choice-set by comparing frequency distributions from varying sampling intensities.

**Fig. 4:**
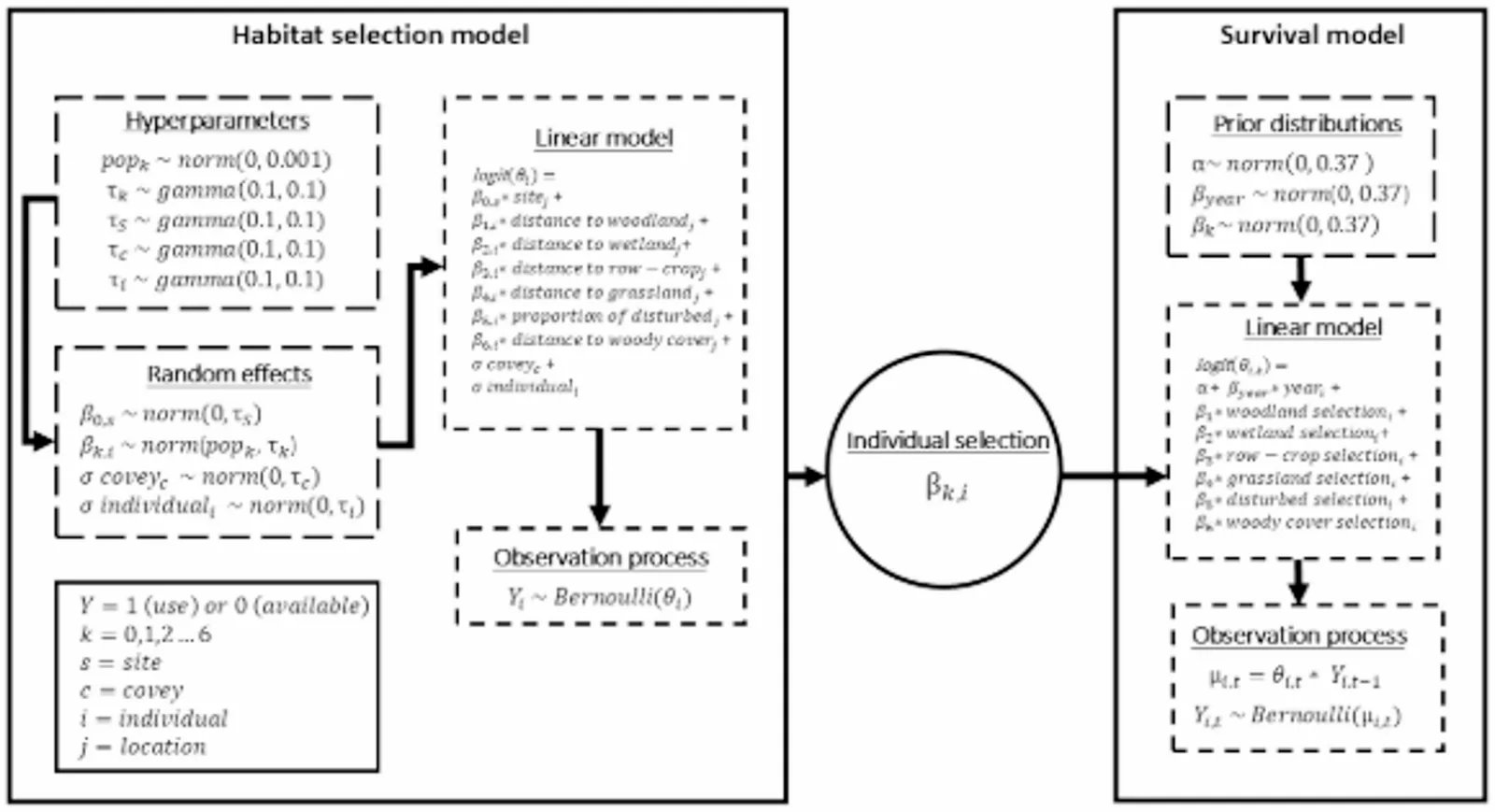
Model descriptions for habitat selection and survival models used to estimate roosting habitat selection and survival rates of northern bobwhite (*Colinus virginianus*) during the fall/winter of 2020–2021 and 2021–2022 in Greene County, Indiana, USA. Mean estimates for nocturnal habitat selection of specific landcover variables for each unique individual (*i*) were derived from the habitat selection model and used as covariates in the survival model to estimate influence of habitat selection on daily survival rates

We used a mixed-effects conditional logistic regression model under a Bayesian framework (Fig. 4) in Jags using the R package “jagsUI” (Kellner 2019) to estimate nocturnal habitat selection. We utilized selected (used) and control (random available) locations to estimate the effects of landcover variables on selection of roost sites. Since landcover variables were spatially autocorrelated within each choice-set, a random effect for unique choice-set was included in our model. We incorporated a random effect for each unique bird to account for variation of selection between individuals in nested groups (Hebblewhite and Merrill 2008; Hill et al. 2023). If a bird occurred in both years’ datasets, then we treated it as two separate individuals since available resources likely varied across years. Due to the social structure of bobwhite during the fall/winter, individuals persist in groups (coveys), where movement/selection rates will be correlated amongst individuals within each specific covey (i.e., if multiple tagged individuals are a member of the same covey, then selection rates will likely be correlated for these individuals). To account for this correlation, we followed Hill et al.’s (2023) approach of assigning a unique grouping (covey) ID for each individual’s location and included it as a random effect. It is important to note that individuals do not always persist in the same covey over time. Many birds in our sample had > 1 covey ID, as we observed covey splitting, amalgamations, and periodic mixings during the duration of our study. Furthermore, number of locations often varied by individual within coveys due to timing of tagging, radio failures, or mortalities. Habitat selection likelihood was described as a Bernoulli distribution (Eq. 1) where *Y* could either equal 0 (available) or 1 (used). Our linear model contained six landcover variables which were used as predictor variables of habitat selection (Eq. 2). Random effects of site, covey, and individual were also in our model and designated as β_0_, σ_covey_, and σ_individual_, respectively (Eq. 2). Each unique individual was represented by *i* while *n* represents the total number of unique birds included in our sample (Eq. 3), *j* corresponds to the specific sampled location (available or used) and *J* equals the number of all sampled locations (Eq. 4), *c* designates the unique covey that an individual was with at the specific location with *C* being the total unique coveys (Eq. 5), and the specific roost site and it’s respective choice-set is distinguished by *s* with *S* representing the total amount of unique roost sites/choice-sets included in our data (Eq. 6).

1 $${Y}_{i} \sim Bernoulli\left({\theta}_{i}\right)$$

2 $$\begin{aligned} logit\left({\theta}_{i}\right) = & \, {\beta}_{{0}_{s}}+{\beta}_{{1}_{i}}*{distance\:to\:woodland}_{j} \\& + {\beta}_{{2}_{i}}*{distance\:to\:wetland}_{j} \\& + {\beta}_{{3}_{i}}*{distance\: to\: {row-}crop}_{j} \\& + {\beta}_{{4}_{i}}*{distance\:to\:grassland}_{j} \\& + {\beta}_{{5}_{i}}*{propotion\:of\:disturbed}_{j} \\& + {\beta}_{{6}_{i}}*{distance\:to\:woody\:cover}_{j} \\& + {\sigma}_{{covey}_{c}} + {\sigma}_{{individual}_{i}} \end{aligned}$$

3 $$i=1, \, 2,\:\dots,\:n$$

4 $$j = 1, \, 2, \:\dots\:,\:J$$

5 $$c = 1, \, 2,\:\dots\:,\:C$$

6 $$s = 1, \, 2,\:\dots\:,\:S$$

To help improve model performance, we scaled all landcover variable data by subtracting the mean from the sampled value and dividing the difference by 1 standard deviation. We assigned a vague normally distributed prior for the hyperparameters used to estimate population level selection for each of our six landcover variables, where *k* corresponds to the unique beta coefficient (Eqs. 7, 8). We also assigned vague gamma distributed priors to estimate variation associated with: landcover selection (Eq. 9), site (Eq. 10), covey (Eq. 11), and individual (Eq. 12).

7 $$\:{pop}_{k}\:\sim\:normal(0,\:0.001)$$

8 $$k=1, \, 2,\:\dots\:,\:6$$

9 $${\tau}_{k}\:\sim\:gamma(0.1,\:0.1)$$

10 $${\tau}_{site}\:\sim\:gamma(0.1,\:0.1)$$

11 $${\tau}_{covey}\:\sim\:gamma(0.1,\:0.1)$$

12 $${\tau}_{individual}\:\sim\:gamma(0.1,\:0.1)$$

Priors for selection of each habitat variable at the individual level were estimated from a normal distribution–the mean and precision terms were set as the estimated hyperparameters of population level selection and variation amongst individuals (Eq. 13). The prior for random effect of site was normally distributed with a mean of zero and used the site variation hyperparameter as the precision term (Eq. 14). Priors for random effects of covey and individual had a normal distribution, mean of zero, and used the hyperparameter of covey and individual variation as precision terms (Eqs. 15, 16).

13 $${\beta}_{k,i}\:\sim\:normal({pop}_{k},\:{\tau}_{individual})$$

14 $${\beta}_{{0}_{s}}\:\sim\:normal(0,\:{\tau}_{site})$$

15 $${\sigma}_{covey}\:\sim\:normal(0,\:{\tau}_{covey})$$

16 $${\sigma}_{individual}\:\sim\:normal(0,\:{\tau}_{individual})$$

To estimate posterior distributions, we generated 3 Markov chain Monte Carlo (MCMC) sets from 20,000 iterations, using 1000 burn-ins, 1000 adaptations, and a thinning rate of 1. Model convergence was determined when the Gelman-Rubin statistic (Gelman et al. 2014), $\:\widehat{R}$, was below 1.1 and by visual inspection of MCMC trace plots. We transformed 95% Bayesian posterior credible intervals (CrI) of beta coefficients to a probability scale for more easily interpreted results. We also report posterior distribution overlap values (PD), which are the proportion of parameter’s estimate that share the same sign as the estimated mean. We interpreted effects to be statistically significant if 95% CrI failed to overlap 0 and used these interpretations to expound on hypotheses.

### Survival analysis

To estimate daily survival rates of bobwhite, we used a known-fates survival analysis under a Bayesian framework (Fig. 4) via “jagsUI” (Kellner 2019). We followed Royle and Dorazio’s (2008) approach of treating fate between successive days as our sampling unit, where the probability of individual *i* surviving from time period *t* to *t* + 1 was conditioned on a Bernoulli distribution (Eq. 17). We designated the observation *Y* as being 1 if an individual *i* was alive at time *t* or 0 if dead. Therefore, if an individual was observed dead at time *t* – 1, the survival probability ϕ at time *t* will equal 0 (Eq. 18). We fit a linear model on the logit scale to examine how nocturnal habitat selection of the six landcover variables from our habitat selection model, as well as year, influenced survival (Eq. 19). We examined correlation values among the habitat selection rates via the Pearson’s correlation test to ensure removal of certain terms wasn’t necessary. Correlation values fell between the range of − 0.44 to 0.49, indicating low to moderate correlation for our covariate data set.

17 $${Y}_{i,t} \sim Bernoulli\left({\mu}_{i,t}\right)$$

18 $${\mu}_{i,t}={\phi}_{i,t}*\:{Y}_{i,t-1}$$

19 $$\begin{aligned} logit\left({\phi}_{i,t}\right) = & \, \alpha + {\beta}_{year}*{year}_{i}+{\beta}_{1}*{\:woodland\:selection}_{i} \\& +{\beta}_{2}*{wetland\:selection}_{i}\\& +{\beta}_{3}*{{row-}crop\:selection}_{i} \\& +{\beta}_{4}*{grassland\:selection}_{i}\\&+{\beta}_{5}*{disturbed\:selection}_{i}\\&+{\beta}_{6}*{woody\:cover\:selection}_{i} \end{aligned}$$

Each land cover selection covariate included in the survival model for individual *i* was the mean selection value derived from the habitat selection model’s posterior distribution for the corresponding bird. All selection covariates were scaled with the same method used for the habitat selection model. Covariate data for year was treated as a dummy variable, where 2020–21 equaled 0 and 2021–22 equaled 1. We used Lunn et al.’s (2012) suggestion of assigning a vague normal distribution with a mean of 0 and precision term of 0.37 for all of our model terms (Eqs. 20–22).

20 $$\alpha\:\sim\:normal(0,\:0.37)$$

21 $${\beta}_{year}\:\sim\:normal(0,\:0.37)$$

22 $${\beta}_{k}\:\sim\:normal(0,\:0.37)$$

Our survival model was run for 50,000 iterations, with 1000 burn-ins and 1000 adaptations, generating 3 MCMC sets which were used to estimate posterior distributions. Convergence was once again determined using the Gelman-Rubin statistic (Gelman et al. 2014) and visual inspection of MCMC trace plots. We present our results as period survival estimates (181 days) to make effects of year and habitat selection on survival more easily interpretable. We used the same approach as mentioned in the habitat selection analysis to determine significance.

## Results

We collected nocturnal habitat use data at 720 unique roost sites (2020–21: *n* = 351, 2021–22: *n* = 369) from 169 radio-tagged bobwhite (2020–21: *n* = 86, 2021–22: *n* = 83). A total of 3 birds were included in both years’ datasets. Due to covey mixing, splitting, and amalgamations, we assigned a total of 70 unique coveys IDs (2020–21: *n* = 37, 2021–22: *n* = 33) to individuals (i.e., an individual may have > 1 covey ID during the study). Mean nocturnal locations recorded per bird were 13.64 $\:\pm\:$ 7.83 (SD) and 14.24 $\:\pm\:$ 8.76 (SD) for 2020–21 and 2021–22, respectively.

Bobwhite which died due to hunting or vehicle collisions during our study were removed from our survival analysis, resulting in a sample size of 132 birds (2020–21: *n* = 63, 2021–22: *n* = 69). Of these 132 individuals, 82 were determined to have suffered mortality; the primary estimated cause being avian predation (avian: *n* = 52, unknown predator: *n* = 16, mammal: *n* = 11, hypothermia: *n* = 2). An additional 16 birds were censored during data collection due to radio-failure, trap related mortalities, or observers failing to relocate individuals after large dispersal events.

### Habitat selection

**Fig. 5:**
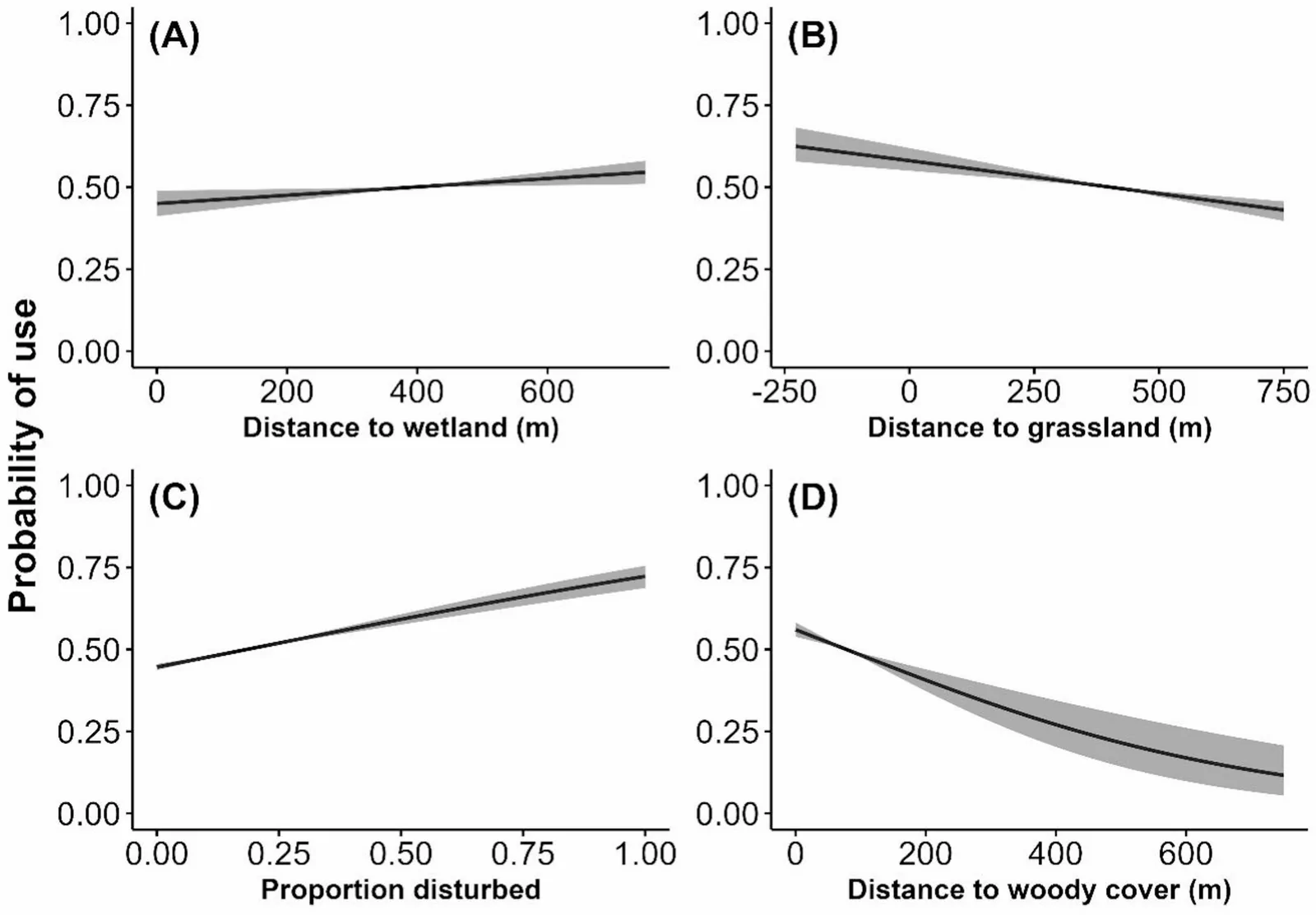
Mean (solid line) and 95% posterior distribution (gray shading) estimates for probability of use as a function of specific land cover metrics for roosting northern bobwhite (*Colinus virginianus*) during the fall/winter of 2020–2021 and 2021–2022 in Greene County, Indiana, USA. Negative distances to grassland (**B**) are interior distance to grassland edge

Results from our roost-site selection model provided some support for our hypothesis of predation risk influencing space use during nocturnal times. The avoidance of areas near wetlands for nocturnal habitat, albeit a weak response, supported the predation risk hypothesis. Our prediction of birds avoiding these areas as nocturnal habitat was supported, as probability of use was 0.45 (95% CrI: 0.41–0.49) at a distance of 0 m to wetlands and increased by a rate of 0.03 (95% CrI: 0.01–0.06) per 250 m increase away from this landcover (Table 1; Fig. 5A). Our prediction of grasslands being selected as roosting habitat was supported, thus providing further support for the predation risk hypothesis. When 228 m inside of grasslands, probability of use was 0.63 (95% CrI: 0.58–0.69) and decreased by a rate of 0.05 (95% CrI: 0.03–0.07) per 250 m increase away from interior areas of grasslands (Table 1; Fig. 5B). Our prediction that disturbed areas would be selected for as nocturnal habitat was supported by our model results, providing more evidence for the predation risk hypothesis (Table 1; Fig. 5C). When proportion of disturbance was 0, probability of use was 0.45 (95% CrI: 0.44–0.46) and increase at rate of 0.06 (95% CrI: 0.05–0.07) per proportion increase of 0.20 (Fig. 5C). The selection of areas near woody cover provided even more support for our predation risk hypothesis. Model results supported the prediction that birds would select roost sites near this protective cover type (Table 1; Fig. 5D). When the distance to woody cover was 0 m, probability of use was 0.56 (95% CrI: 0.54–0.58) and decreased at rate of 0.19 (95% CrI: 0.17–0.21) per increasing distance of 250 m. We observed no effect of row-crops on nocturnal habitat selection as 95% posterior distributions overlapped 0 (Table 1), failing to provide support for our prediction that roosting birds would avoid this cover type. The relatively weak mean beta coefficient estimates for the effect of row-crops also suggest that it is not influential regarding nocturnal habitat selection (Table 1). We found no support for our prediction of birds avoiding areas near woodlands for nocturnal habitat selection as 49% of the posterior distribution overlapped zero (Table 1). Moreover, the mean effect for our beta coefficient was centered on 0 (Table 1).

**Table 1:** Model coefficient posterior estimates (logit scale) for all land cover covariates included in a nocturnal habitat selection model for northern bobwhite (*Colinus virginianus*) during the fall/winter in Greene County, Indiana, USA, 2020–2021 and 2021–2022. PD represents the proportion of the posterior distribution that fails to overlap 0

Habitat selection	$\stackrel{-}{\boldsymbol{x}}$	SD	Credible intervals		PD
Covariate			2.5%	97.5%	
Woodland	0.00	0.05	− 0.10	0.10	0.52
Wetland*	0.18	0.07	0.04	0.32	0.99
Row-crop	− 0.07	0.04	− 0.16	0.01	0.96
Grassland*	− 0.51	0.11	− 0.77	− 0.33	1.00
Disturbed*	0.43	0.04	0.36	0.50	1.00
Woody cover*	− 0.19	0.04	− 0.26	− 0.12	1.00
Deviance	18,952.78	29.59	18,893.53	19,009.72	1.00

*Indicates significant effect

### Survival

**Fig. 6:**
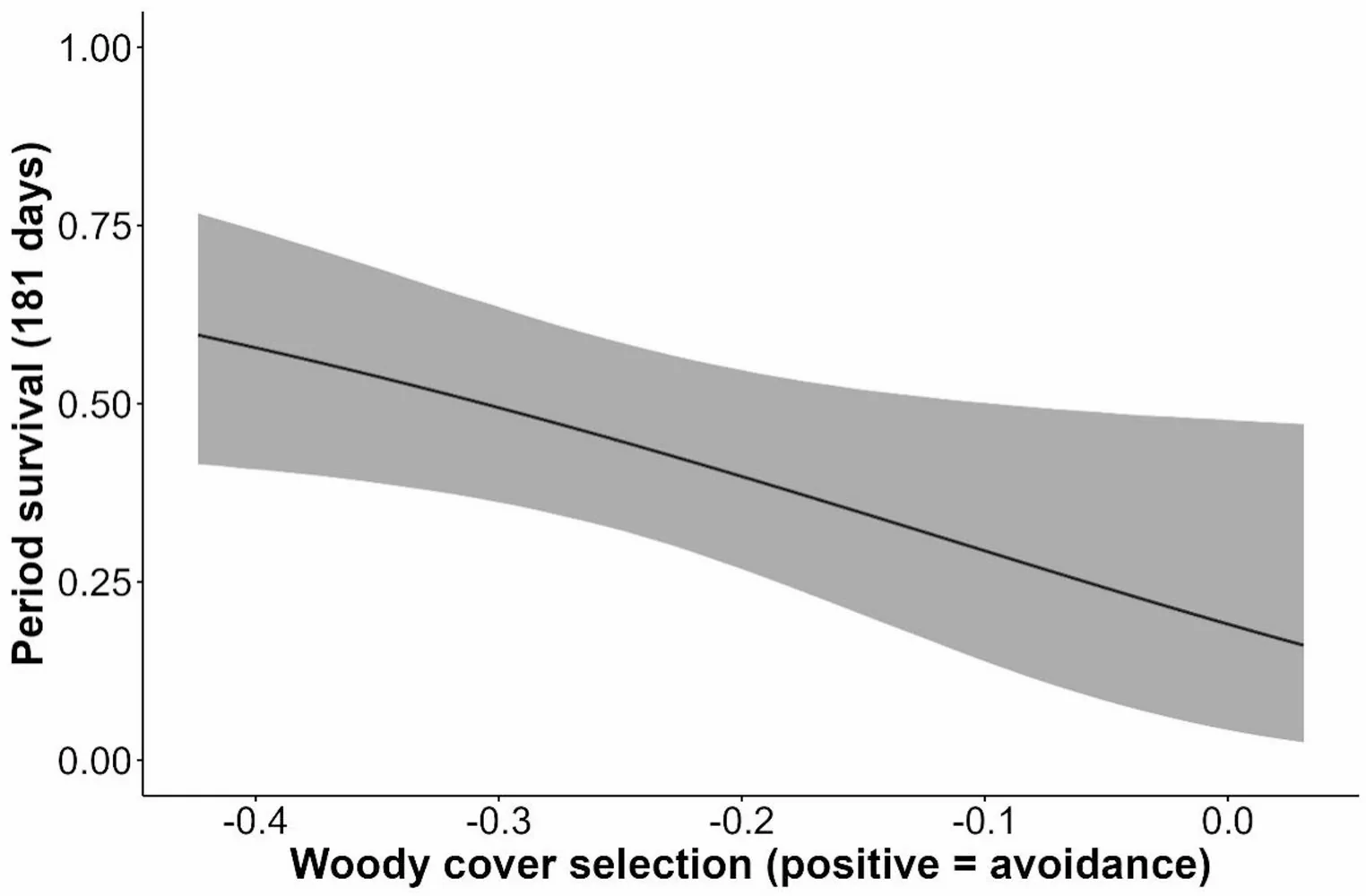
Mean (solid line) and 95% posterior distribution (gray shading) estimates for period survival (181 days) as a function of woody cover selection for roosting northern bobwhite (*Colinus virginianus*) during the fall/winter of 2020–2021 and 2021–2022 in Greene County, Indiana, USA

Mean period survival (181 days) did trend slightly higher for year 2 ($\stackrel{-}{x}=0.40$) relative to year 1 ($\:\stackrel{-}{x}=0.31$), but no statistically significant effect of year was observed between estimates since posterior distributions had considerable overlap between the two cohorts (Year 1 95% CrI: 0.21–0.46; Year 2 95% CrI: 0.27–0.55; Table 2). The observed positive effect between woody cover selection and survival provided supporting evidence for the ideal free distribution hypothesis (Table 2; Fig. 6). Mean period survival estimates were 0.60 (95% CrI: 0.42–0.77) for individuals with the strongest selection for woody cover (− 0.42); survival decreased by approximately 0.13 (95% CrI: 0.11–0.15) for every 0.10 unit increase in selection (i.e., more negative selection value equates to stronger preference for woody cover; Fig. 6). These results were congruent with our prediction that birds selecting roost sites closer to woody cover would have greater survival rates relative to individuals that had weak/no preference for this cover type. No support was provided for the ecological trap hypothesis as we failed to observe a negative effect on survival for all other landcover selection rates (Table 2).

**Table 2:** Model coefficient posterior estimates (logit scale) for year and nocturnal habitat landcover selection rate covariates included in a survival model for northern bobwhite (*Colinus virginianus*) during the fall/winter in Greene County, Indiana, USA, 2020–2021 and 2021–2022. PD represents the proportion of the posterior distribution that fails to overlap 0

Survival	$\stackrel{-}{\boldsymbol{x}}$	SD	Credible intervals		PD
Covariate			2.5%	97.5%	
Intercept/slope	5.10	0.18	4.76	5.46	1.00
Year effect	0.21	0.30	− 0.38	0.80	0.76
Woodland	− 0.18	0.11	− 0.38	0.03	0.95
Wetland	0.03	0.14	− 0.25	0.30	0.59
Row-crop	0.12	0.12	− 0.12	0.37	0.83
Grassland	− 0.02	0.15	− 0.30	0.27	0.56
Disturbed	− 0.14	0.11	− 0.36	0.07	0.90
Woody cover*	− 0.27	0.13	− 0.54	− 0.02	1.00
Deviance	1,001.85	3.92	996.11	1011.15	1.00

*Indicates significant effect

## Discussion

We found considerable supporting evidence for the predation risk hypothesis via roosting birds avoiding areas adjacent to wetlands, selecting grasslands and disturbed areas, as well as selecting sites near woody cover. The lack of effect for row-crop fields and woodlands on roost-site selection provided no supporting or opposing evidence for the predation risk hypothesis. We tested whether habitat selection influenced survival to evaluate IFD and ecological trap hypotheses. Our observation that woody cover selection improved survival provided one instance of IFD functioning in this system, suggesting that predation risk is likely the driving force behind this process. We were unable to provide any further evidence of IFD occurring, as well as no supporting evidence for ecological traps.

### Roost site selection supports ideal free distribution

We found support for the IFD hypothesis through the positive relationship associated with woody cover selection and survival. This result illustrates the importance of woody cover for bobwhite during the fall/winter, as survival benefits were apparent for birds that exhibited strong selection for this cover type.

Limited dispersion of woody cover throughout the landscape could be a limiting factor with regards to habitat quality during the fall/winter period at sites similar to ours, where woody cover is often narrow, linear features. Providing woody cover at a rate which mitigates variation in selection at the population level (i.e., woody cover is dispersed at regular intervals throughout occupiable space) could help bolster survival rates of northern bobwhite, particularly on sites where it is limited. Overall, our results illustrate that woody cover offers a critical resource regarding predator refuge, however, the degree to which it benefits bobwhite survival likely depends on the amount and spatial arrangement of this habitat component.

While our habitat selection results show that interior portions of grasslands and disturbed areas are strongly selected for as nocturnal habitat, we observed no effect on survival. One explanation could be that suitable patches of habitat which are conducive for predator evasion and vigilance are distributed uniformly throughout these cover types. In turn, predation would seemingly occur at random—thus resulting in the lack of an effect on survival. Singh et al.’s (2012) study on bobwhite nesting ecology drew similar conclusions, as they hypothesized that predation likely occurred at random due to widespread availability of suitable habitat. This trend has also been observed in other systems where influence on fitness by resource selection were likely mediated by abundant and widely distributed high-quality resources (Long et al. 2016).

### Predation risk drives roost site selection

Our results provided support for the hypothesis that predation risk is a driver of nocturnal habitat selection, aligning with previous studies on bobwhite (Chamberlain et al. 2002; Hiller and Guthery 2005; Perkins et al. 2014; Hill et al. 2023) and other Galliformes (Tillman 2009; Xu et al. 2010; Yuan et al. 2018; Adey et al. 2023), many of which have concluded that risk is reduced by vegetation structure which enables concealment and predator evasion. Selection of herbaceous-dominated cover types (grasslands and disturbed areas) aligned with previous works (Wiseman and Lewis 1981; Chamberlain et al. 2002). Within these areas, vegetation structure likely balances vigilance and concealment, while limited canopy closure would provide birds with unhindered flight paths for flushing during predator encounters. Additionally, strong selection for areas near woody cover depicts that access to additional refuge is impactful, as this cover type provides protection from predators (Yoho and Dimmick 1972; Williams et al. 2000; Perkins et al. 2014; Sinnott et al. 2022). Therefore, it appears that roost site selection is impacted by both vegetation structure at roost sites and the distance from additional escape cover.

Roost site selection may also be influenced by predator distributions, as birds avoided areas near wetlands and selected interior portions of grasslands and disturbed areas. These behaviors reflect avoidance of cover types with high densities of nocturnal predators (Fritzell 1978; Arnold and Fritzell 1990; Newbury and Nelson 2007) and landscape features that are associated with predator activity centers and travel corridors (Hinton et al. 2015; Deuel et al. 2017; Abouelezz et al. 2018). Row-crop fields had no influence on habitat selection, lending no supporting or opposing evidence for the predation risk hypothesis. Although these fields provide readily accessible food for bobwhite, vegetation characteristics and predator avoidance likely have a greater influence on roost site selection (Thompson et al. 2009; Adey et al. 2023).

It is possible that the scale at which we quantified selection and our modeling approach limited our ability to detect effects on survival. Notably, our study did not quantify microsite characteristics and fourth-order selection at roost sites, which may impact fitness. Additionally, we did not model selection as time-varying, which could be fluid over time conditioned on predator interactions. Pertaining to our survival model, using mean selection rates as individual covariates does not account for uncertainty associated with habitat selection estimates. However, we have minimal concern with our interpretation of woody cover selection improving survival, given the strong signals in both nocturnal selection and effects on survival. Future studies on bobwhite roost site selection should investigate temporal variation in selection and examine if similar patterns exist across different life stages (e.g., brood rearing) or in other landscapes. Furthermore, utilizing hierarchical approaches that account for uncertainty associated with individual selection rates could provide more robust estimates of the links between selection and fitness, although such methods may be computationally burdensome. Lastly, manipulative experiments that alter habitat components or resources will be vital for identifying causal links between resources and demographic parameters (Reynolds-Hogland et al. 2008; Malone et al. 2022).

## Conclusions

Our results indicate that predation risk strongly shapes nocturnal habitat selection of bobwhite and that woody cover selection improves survival, providing support for ideal free distribution and no evidence for ecological traps. These findings highlight the value of linking selection to fitness, as selection of predator refugia conferred direct impacts on fitness. Implementing conservation practices that are exclusively based on resource/habitat selection results can be problematic because selection and fitness are not always correlated (Dwernychuk and Boag 1972; Gates and Gysel 1978; Delibes et al. 2001; Donovan and Thompson 2001). To ensure management recommendations are focused on promoting beneficial resources for the species of interest, studies should investigate if, and to what degree, resource selection impacts a species’ demographic rates. We believe that future research conducted on resource/habitat selection of animals which are directly observed (i.e., via GPS or radio-tagging) should incorporate a survival component to the modeling approach. Information ascertained from research with a similar approach as we have illustrated will help develop more comprehensive understandings of complex systems, and how vital rates of individuals in those populations are influenced by resources in their environments. Although integrated approaches that link selection to fitness are rare, they provide practical information on how resource use influences fitness. Moreover, expanding this approach across a wide array of species and ecosystems will provide more effective, demographically informed conservation practices.

## Data Availability

All associated habitat selection and survival data are publicly available through Dryad Digital Repository: https://doi.org/10.5061/dryad.wpzgmsc54.

## Code availability

Code to conduct habitat selection and survival analyses are publicly available through Dryad Digital Repository: https://doi.org/10.5061/dryad.wpzgmsc54.
